# The MedXFit-study – CrossFit as a workplace health intervention: a one-year, prospective, controlled, longitudinal, intervention study

**DOI:** 10.3389/fpubh.2024.1304721

**Published:** 2024-02-21

**Authors:** Tom Brandt, Elisabeth Heinz, Yannik Klaaßen, Selina Limbara, Marian Mörsdorf, Timo Schinköthe, Annette Schmidt

**Affiliations:** ^1^Institute of Sports Science, University of the Bundeswehr Munich, Neubiberg, Germany; ^2^Comprehensive Cancer Center Munich CCCLMU, Munich, Germany

**Keywords:** behavioral change and maintenance, functional fitness, mobility, strength, well-being, back pain, military, exercise

## Abstract

**Introduction:**

Workplace health interventions aim to motivate employees toward healthy behaviors to improve fitness and health in the long-term. We investigated whether CrossFit® is an effective training concept to achieve these goals in inactive employees with sedentary occupations.

**Methods:**

The study followed a prospective, controlled intervention design. Employees were invited to participate in intervention group (IG) or control group (CG) on their own preferences. Inclusion criteria were a predominantly sedentary occupation and execution of less than two muscle and/or mobility enhancing training sessions per week at the time of enrolling. The IG did at least two times a week a CrossFit training of 1 h. Mobility, strength, well-being, and back-issues were measured at the beginning, after 6, and 12 months. Participants in the CG were free to choose any other activities offered at the same time (e.g., circuit training, meditation, full body stability training). Adherence, respectively, behavioral change and maintenance qualities were evaluated based on the COM-B system and presence of behavior maintenance motives.

**Results:**

89 employees were enrolled into the trial, from where 21 dropped out due to external factors (24%). From the remaining participants, 10 out of 39 (26%) in the IG and 1 out of 29 (4%) in the CG stopped for intrinsic reasons, leading to a non-adherence to the intervention of 22 percentage points. Motivation for behavioral change and maintenance in the IG was primarily driven by enhanced physical and psychological capability. Development of physical capability was evident by significant improvements (*p* < 0.001) in the IG compared to the CG for mobility (*d* = 3.3), maximal isometric strength (min. *d* = 1.7, max. *d* = 2.5), as well as reduction in pain intensity (*p* = 0.003, *r* = 0.4) and frequency (*p* = 0.009, *r* = 0.35) after 12 months. Significant improvements between the 6-month and the 12-month measurement in mobility and 6 out of 8 strength measures within the IG indicated the effectiveness of CrossFit beyond the beginner phase.

**Conclusion:**

CrossFit is a motivating training concept that led to long-term health and fitness improvements in inactive employees doing sedentary work and should be given greater consideration in workplace health promotion.

## 1 Introduction

Physical inactivity and sedentary behavior are key risk factors regarding the development of non-communicable diseases and classified as a worldwide issue (1–3). Given the high prevalence of predominantly sedentary occupations in modern society, workplace health promotion (WHP) provides great potential to reach the most vulnerable clientele (4, 5). Cost-effectiveness and health benefits of WHP were demonstrated in several previous studies (6–9). But although the workplace offers efficient structures to reach large groups and makes use of a natural social network, participation levels in WHP were typically below 50% (10–12).

Behavioral change frameworks can help to design and evaluate WHP interventions (13). An applicable framework for this purpose is provided by the COM-B system. According to the COM-B system there are three main factors that influence behavior – capability, opportunity, and motivation. Capability is understood as the individual’s physical and psychological capacity to execute an activity. Motivation involves those brain processes that stimulate and control behavior such as habitual processes, emotional responding, and conscious decision-making. Opportunity represents all factors external to the individual that enable the behavior or trigger it. Because capability and opportunity both impact the motivation to show a certain behavior, manipulating them can initiate behavioral change. Performing a certain behavior can in turn affect opportunity, capability, and motivation (13).

Due to its health-focused approach and high scalability, an auspicious training concept for unfit, sedentary individuals may be provided by CrossFit® (*CF*). *CF* is a functional fitness program that emphasizes broad fitness adaptations. Characteristic about *CF* is the integration of complex compound movements from different sports (e.g., gymnastics, powerlifting, weightlifting, kettlebell lifting) in order to improve strength, coordination, and mobility (14). This aspect could be particularly relevant for WHP, given the high prevalence of sickness absence in the workplace caused by musculoskeletal disorders (15).

Previous studies on *CF* showed positive health related physiological (e.g., body composition, cardiovascular/respiratory fitness, strength, flexibility, power, and balance) and psychological effects as well as injury rates comparable to that in Olympic weightlifting, basic weightlifting, and gymnastics (3.24 injuries / 1,000 h of trainings) (16–21). However, to date studies with high level of evidence and low risk of bias are sparse (16). Data regarding long term effects of *CF* on musculoskeletal fitness and well-being in physically inactive employees does not exist. In consequence, we conducted the MedXFit-study. After 6 months of *CF* training, we found large positive effects for mobility (Functional Movement Screen Score, *p* < 0.001, ⴄ^2^ = 0.58) and maximum isometric strength measures (Dr. Wolff BackCheck®, *p* < 0.001, minimum ⴄ^2^ = 0.18, maximum ⴄ^2^ = 0.47) in *CF* beginners (22). While these initial improvements appeared promising, additional research still had to show that *CF* is effective beyond the beginner level and encourages employees to maintain the newly adopted behavior. Therefore, we conducted a follow-up study for another 6 months with the same study population to investigate long-term effects of *CF.*

In conclusion, we aimed to evaluate how *CF* affects participants’ capability, opportunity, motivation, and behavior. It must be considered that the University of the Bundeswehr Munich (UniBw M) already provided its employees great opportunity for participation in physical activity before the MedXFit-study. Employees were allowed to train for 90 min per week during working hours. They could choose from a broad course program including yoga, circuit training, meditation, functional fitness training, full body stability training, or athleticflow and had access to several training facilities (e.g., swimming pool, climbing hall, fitness center, tennis courts, outdoor fitness park). All facilities and courses were on campus and free of charge. Although this should have facilitated participation in some form of physical activity, high numbers of employees remained inactive until the MedXFit-study (13). Therefore, besides changes in fitness measures, we examined what differentiated *CF* training from other WHP interventions in terms of behavioral change and maintenance.

The trial was registered on ClinicalTrials.gov with the trial number NCT05109286.

## 2 Materials and methods

### 2.1 Trial oversight

The MedXFit-study was a prospective, longitudinal intervention study with control (CG) and intervention group (IG) conducted at the University of the Bundeswehr Munich (UniBw M). Data were collected from October 2020 to March 2022. While the IG participated in 2 CrossFit® (*CF*) trainings per week for 12 months, the CG was free to attend any other activities offered at the same time (e.g., circuit training, meditation, full body stability training). Training sessions of the IG were conducted at the military affiliate *CF* Kokoro® and led by certified *CF* level 1 and 2 coaches. Both groups were tested at baseline (t0), after 6 (t1), and 12 months (t2). To enable participation during working hours the study was integrated in the workplace health promotion (WHP) of the UniBw M.

**Figure 1:**
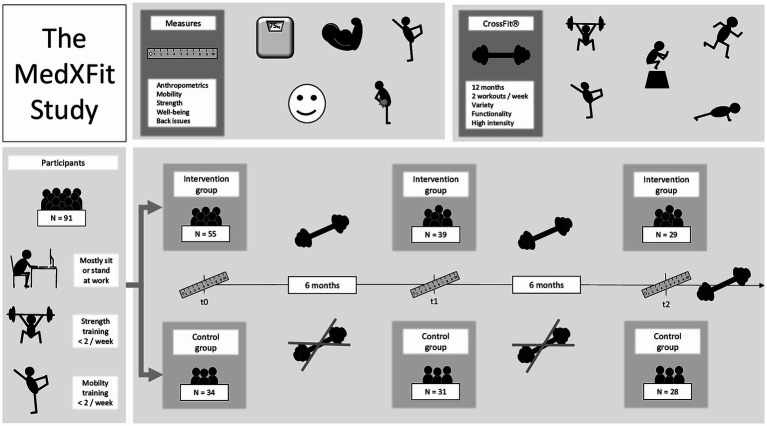
Schematic overview of the MedXFit-study adapted from Brandt et al. (22).

The study was carried out according to the guidelines of the Declaration of Helsinki and approved by the Institutional Ethics Committee of the UniBw M (06/04/2018) and informed consent was obtained from all participants involved in the study. The trial was registered on ClinicalTrials.gov with the trial number NCT05109286. The main aspects of the study design are summarized in Figure 1. A comprehensive study protocol can be found in Supplementary Table S1.

### 2.2 Participants

Military and civilian staff of the UniBw M aged 18–65 years participated in the study. Inclusion was reserved to individuals with a predominantly sitting or standing occupation that did <2 muscle and / or mobility enhancing training sessions per week prior the study. Detailed inclusion and exclusion criteria can be found in Supplementary Table S1. Baseline demographic and anthropometric data of participants were already published by Brandt et al. (22).

### 2.3 CrossFit training

*CF* training was offered at the military affiliation CrossFit Kokoro, Neubiberg, Germany. Programming was done according to the standards from the *CF* Level 1 training guide (14), *CF* Course Planning, and *CF* Scaling course. Every session was concepted for 60 min in classes up to 10 participants. Planning and coordination were done by certified coaches (*CF*-L1 or *CF*-L2 certification). During COVID-lockdown training was consistently possible, but offered in addition as online-classes for those employees who were working from home office.

### 2.4 Study endpoints and protocol

Adherence was measured to discuss *CF*’s potential for behavioral change and maintenance based on the COM-B framework (13). Changes in mobility [Functional Movement Screen (23, 24)] were primary endpoints. Secondary endpoints were changes in strength [maximum isometric strength in kg; Dr. WOLFF BackCheck® 617 (25, 26)] and well-being [WHO-5 score (27)]. Back-issues (pain intensity, perceived limitation, and pain frequency) were assessed by questionnaire for exploratory purpose at t0, t1, and t2.

All test sessions were conducted according to the same study protocol. At first, medical history, physical activity, well-being, and back-issue data were collected via questionnaire. Subsequently, anthropometrics, mobility, and strength measures were taken. Participants executed all tests in sportswear without shoes and avoided intensive physical activities 24 h before the test sessions. Due to COVID-19 pandemic, participants wore a breathing mask during the test sessions.

#### 2.4.1 Adherence

Adherence was calculated by dividing the number of remaining participants by the sum of remaining participants and participants that dropped out intrinsically motivated [adherence = N_remaining_ / (N_remaining +_ N_intrinsic dropout_)]. Non-adherence was defined as the difference between the adherence of CG and IG (non-adherence = adherence_CG_ - adherence_IG_).

#### 2.4.2 Body composition

Bodyweight and height were measured with a TANITA® BC-545 and SECA® 213 scale. Both measures were prerequisite to conduct strength tests with the Dr. WOLFF BackCheck® 617.

#### 2.4.3 Mobility

Mobility was assessed via Functional Movement Screen (FMS) (23, 24). Participants executed 7 fundamental movements (deep squat, hurdle step, inline lunge, shoulder mobility, active straight leg raise, trunk stability push up and rotary stability quadruped). Each movement was done slowly for 3 times in a row whereby the best attempt was counted. Quality of movement execution was rated on a scale from 1 to 3, but a score of 0 was given if the participant reported any pain. The shoulder mobility, trunk stability, and rotary stability quadruped test were followed by a clearing test. Pain during the clearing test resulted in a score of 0 in the corresponding movement. In case of bilateral movements, the lower scored side was counted, which resulted in a maximum score of 21 (23, 24).

#### 2.4.4 Strength

Maximum isometric strength (in kilograms) was measured with the Dr. WOLFF BackCheck® 617 (BC). Criteria validity and test / retest reliability of the BC are sufficient to be applied in scientific research (26). Participants did 3 attempts of the following movements: trunk extension (TE), trunk flexion (TF), upper body push (UPush), upper body pull (UPull), trunk lateral flexion left (TLFl) and right (TLFr), and hip extension left (HEl) and right (HEr). The best attempt per movement was selected for analysis.

#### 2.4.5 Well-being

Well-being was assessed with the World Health Organization Well-Being Index (WHO-5). It is a time efficient tool that offers adequate validity and simplicity (27). The questionnaire is composed of 5 questions about subjective well-being, which are rated on a 6-point scale (0 = worst, 5 = best) resulting in a total score of 0–25.

#### 2.4.6 Back-issues

Issues in the neck, shoulders, upper back, and lower back were separately assessed via questionnaire. Participants were asked whether they experienced issues in these areas during the past 6 months and rated their average pain intensity and perceived limitation on an 11-point scale (0 = no pain or limitation, 10 = highest imaginable pain or limitation). Further, participants reported the number of days per week suffering from back-issues in these areas. For pain intensity, limitation, and frequency the area with the highest score was included in the analysis.

### 2.5 Statistical approach

Participants did insufficient muscle and / or mobility enhancing training before the intervention. Based on improvements in the IG after the first 6 months of *CF* training and previous studies confirming high FMS scores among *CF* athletes compared to inactive individuals we assumed large effects for change in mobility from t0 to t2 and medium effects for t1 to t2 (22, 28–31). Consequently, 27 participants per group were determined to achieve a power of at least 85% on a one-sided 5% significance level. With an expected dropout similar to the first 6 months of the MedXFit-study (IG = 29%, CG = 9%), enough participants of the IG (*N* = 39) and CG (*N* = 31) were willing to continue the study (22).

Difference in change between groups was analyzed to determine the effectiveness of the intervention. Therefore, change values of IG and CG were calculated by subtracting t0 from t2 values as well as t1 from t2 values. Normal distribution was analyzed with Q-Q-plots and Kolmogorov–Smirnov test. Regarding mobility and strength, an independent t-test was conducted to analyze the difference in change values between IG and CG. The effect size is given as Cohen’s d. Differences in change between groups for well-being and back-issue data were analyzed with Mann–Whitney-U test. As suggested by Fritz et al. (32), the effect size r was calculated by dividing the z-value of the Mann–Whitney-U tests by the square root of the sample size N. Statistical significance was set at *p* ≤ 0.05. Mobility, strength, well-being, and back-issue values at t0, t1, and t2 are expressed as mean (SD). Difference in change between groups is given as mean [95% CI]. Data analysis was done with SPSS 28® (IBM SPSS, Armonk, NY, USA).

## 3 Results

### 3.1 Adherence

Eighty-nine employees were enrolled into the MedXFit-study [intervention group (IG): *N* = 55, control group (CG): *N* = 34]. In total, 47% of the IG and 18% of the CG dropped out over the course of 12 months due to lacking intrinsic motivation and various external reasons (e.g., relocation, health issues, parental leave, extended business trips as well as switching to part time or remote work that did not allow to adhere to the training program). During the first 6 months of the study, the IG lost 15 participants of which 9 quit for intrinsic and 6 for extrinsic reasons, resulting in an adherence of 82% [adherence = N_remaining_ / (N_remaining +_ N_intrinsic dropout_)]. Of those that dropped out after 6 months of *CF* training, 1 participant mentioned intrinsic and 9 extrinsic reasons. Adherence of the IG after 12 months reached 74%. With 1 intrinsic and 1 extrinsic motivated dropout during the first 6 months, the CG achieved an adherence of 97%. After 12 months, another 4 participants of the CG left for extrinsic reasons, leading to an adherence of 97%. This resulted in a non-adherence to the intervention of 15 percentage points after 6 months and 22 percentage points after 12 months.

**Figure 2:**
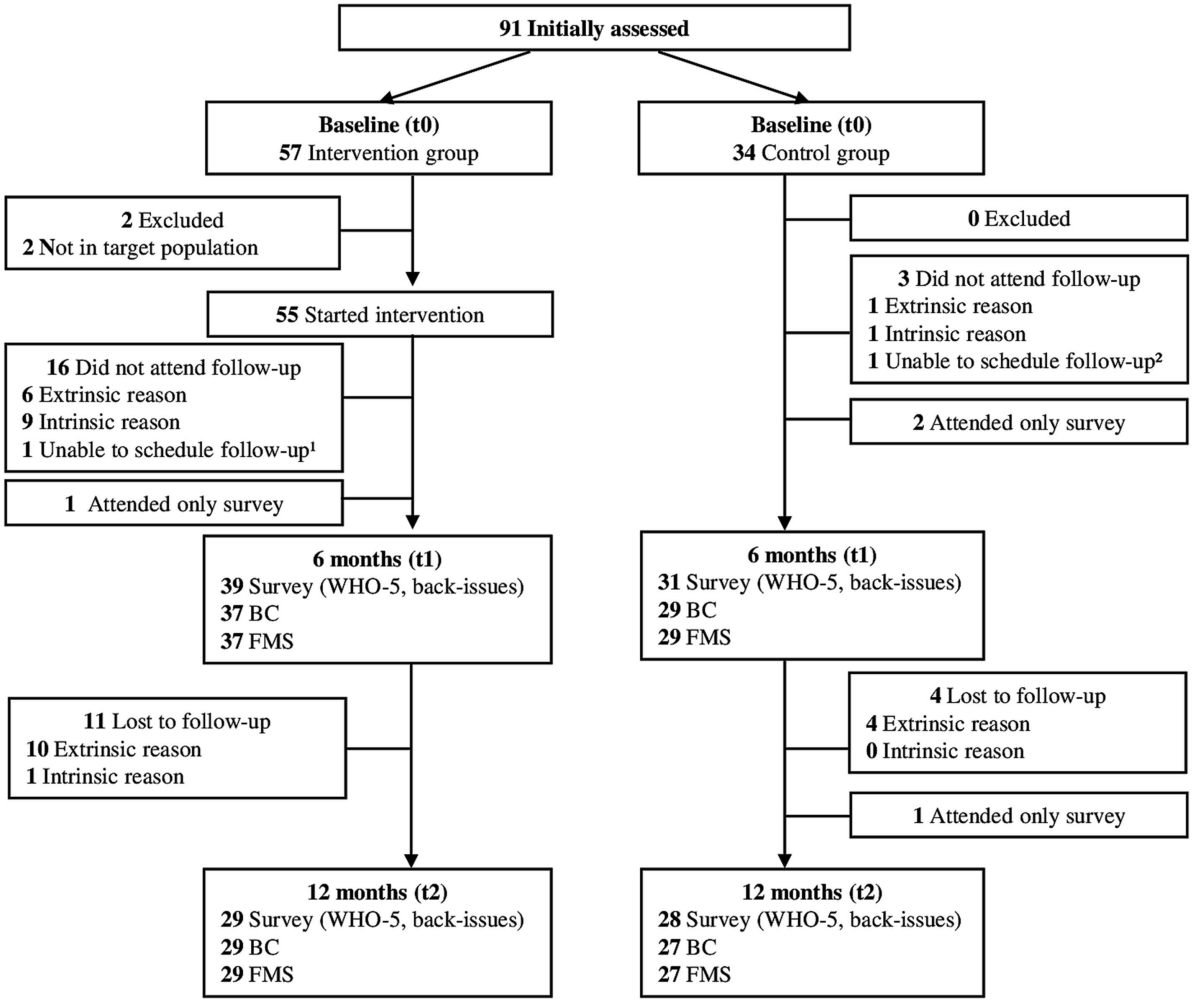
Participant flow over the course of the study.

In addition to those that did not complete all measures at t1 there was another participant of the CG not being able to attend test session t2 in person but per telephone. Furthermore, 1 participant of the CG had to leave out upper body push (UPush) and upper body pull (UPull) in the BackCheck (BC) at t2 due to a minor shoulder injury. Over the course of 52 weeks, participants of the IG attended on average 79.3 (19.3) training sessions. The participant flow is displayed in Figure 2.

### 3.2 Primary endpoint

**Figure 3:**
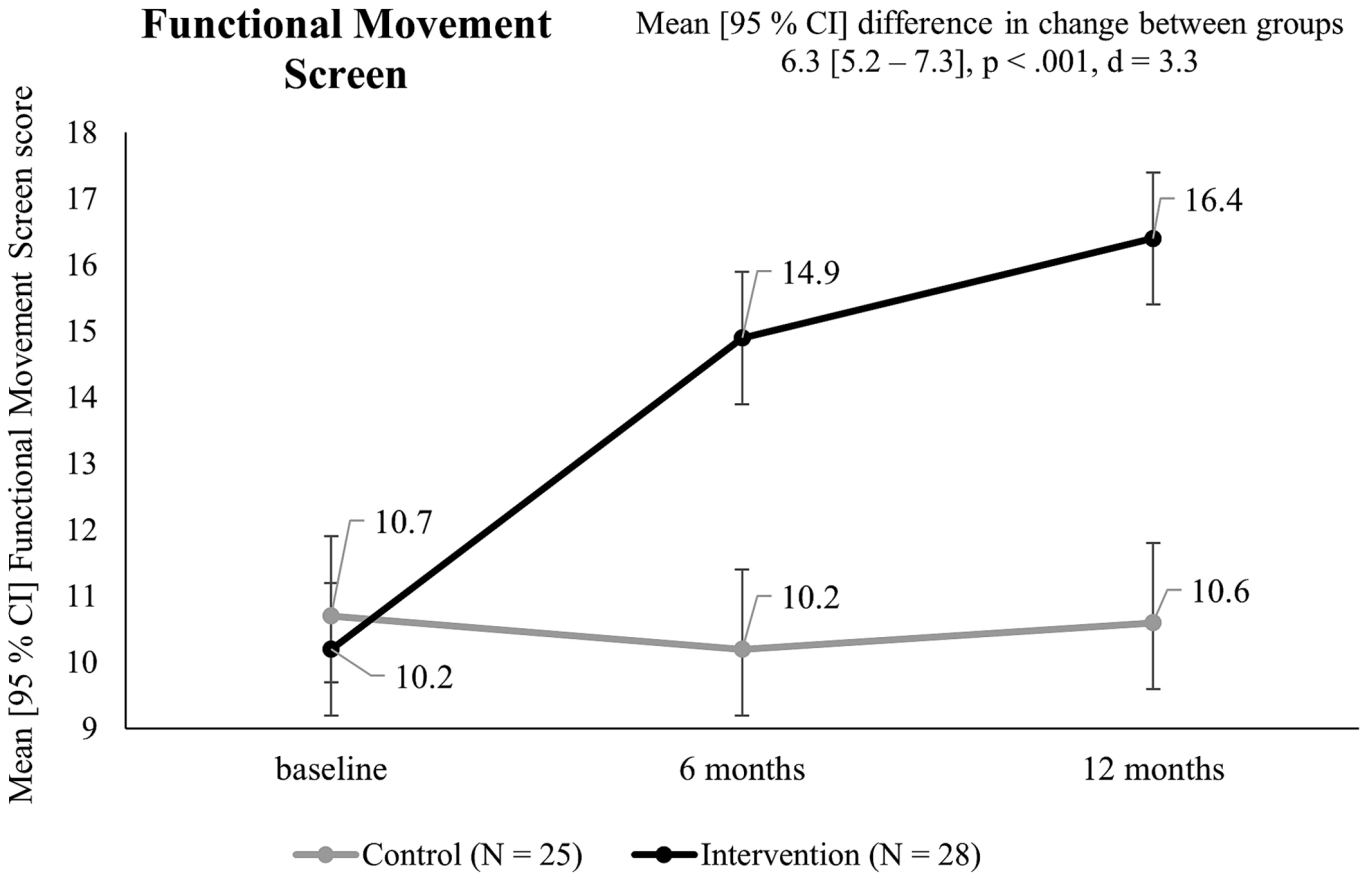
Functional Movement Screen scores in the intervention and control group over the course of 12 months.

Groups did not differ significantly in mobility at baseline (*p* = 0.49, *d* = 0.19). Over the course of 12 months, a significant difference in change in FMS scores between groups of 6.3 [5.2–7.3] was observed (*p* < 0.001, *d* = 3.3). Although change between groups diminished from t1 to t2, a significant improvement in the IG compared to the CG was found (*p* < 0.001, *d* = 0.63). Before the intervention, there was 1 participant in each group that reached a score > 14. After 12 months, 6 participants of the IG scored ≤14 (2 of them with a score of 14), while in the CG all participants scored ≤14 in the FMS (4 of them with a score of 14). Figure 3 illustrates the change over time for both groups.

### 3.3 Secondary endpoints

**Figure 4:**
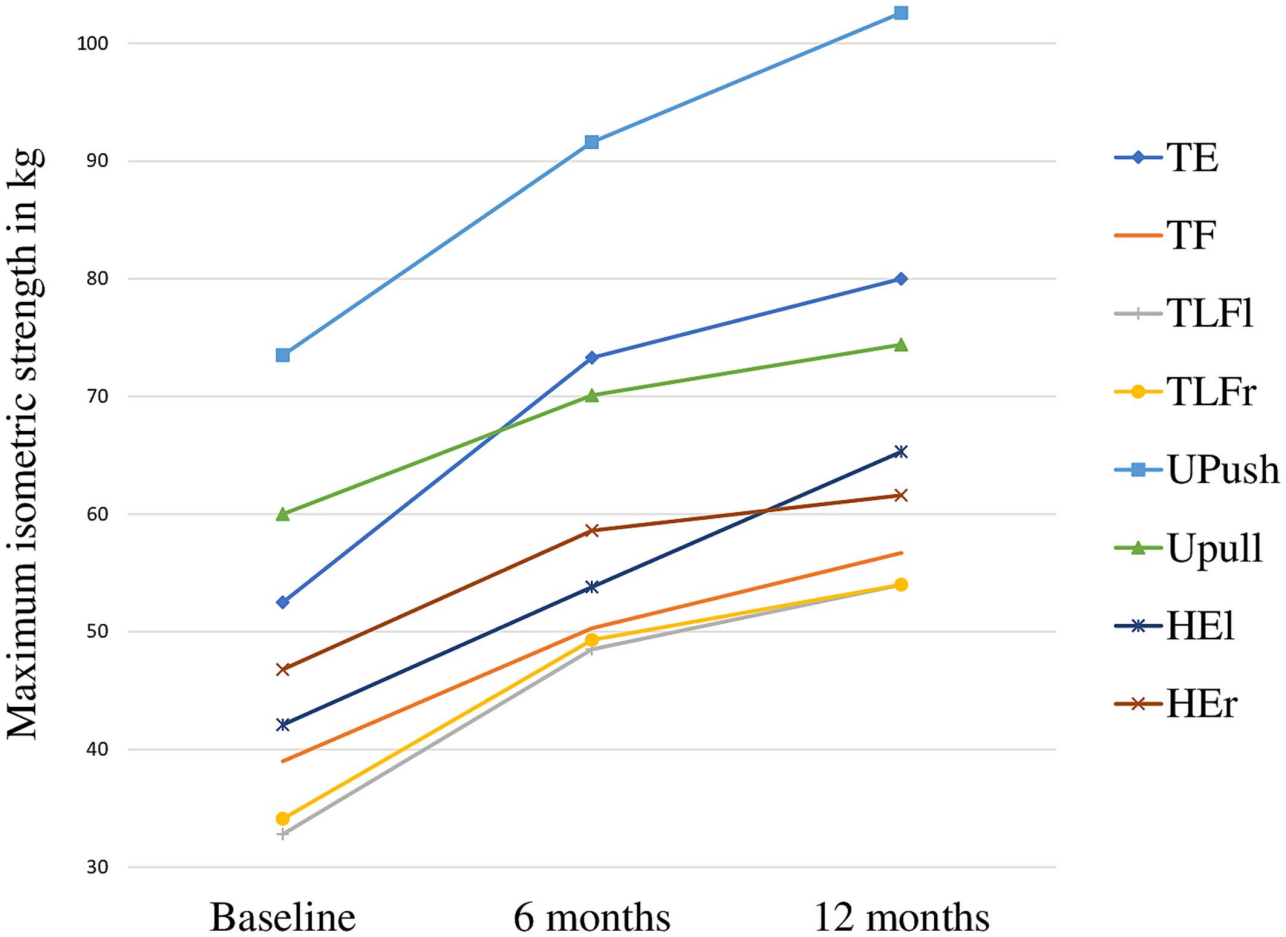
Maximum isometric strength in the intervention group over the course of 12 months. TE, trunk extension; TF, trunk flexion; TLFl, trunk lateral flexion left; TLFr, trunk lateral flexion right; UPush, upper body push; UPull, upper body pull; HEl, hip extension left; HEr, hip extension right.

At baseline, the IG showed higher maximum isometric strength values compared to the CG (minimum *d* = 0.21, maximum *d* = 0.41), but non reached statistical significance. While the IG improved their baseline maximum isometric strength across all strength tests (TE: 24%, TF: 45%, TLFl: 65%, TLFr: 58%, UPush: 40%, UPull: 24%, HEl: 55%, and HEr: 32%; (t2 – t0) / t0) after 12 months, negative and positive change occurred in the CG (TE: 5%, TF: -6%, TLFl: 12%, TLFr: 13%, UPush: 4%, UPull: 2%, HEl: 16%, and HEr: −5%, (t2 – t0) / t0). Difference in change between groups was significant for all strength tests (*p* < 0.001, *d* ≥ 1.7). The largest effects occurred for trunk flexion (*d* = 2.5), trunk lateral flexion left (*d* = 2.3), and trunk extension (*d* = 2.3). Between t1 and t2 significant improvements in the IG compared to the CG occurred for TE (*p* = 0.009, *d* = 0.75), TF (*p* < 0.001, *d* = 0.79), TLFl (*p* = 0.018, *d* = 0.67), UPush (*p* = 0.008, *d* = 0.77), UPull (*p* = 0.004, *d* = 0.84), and HEl (*p* < 0.001, *d* = 1.31), but not for TLFr (*p* = 0.32, *d* = 0.28) and HEr (*p* = 0.184, *d* = 0.37). Strength developments of the IG are presented in Figure 4.

**Figure 5:**
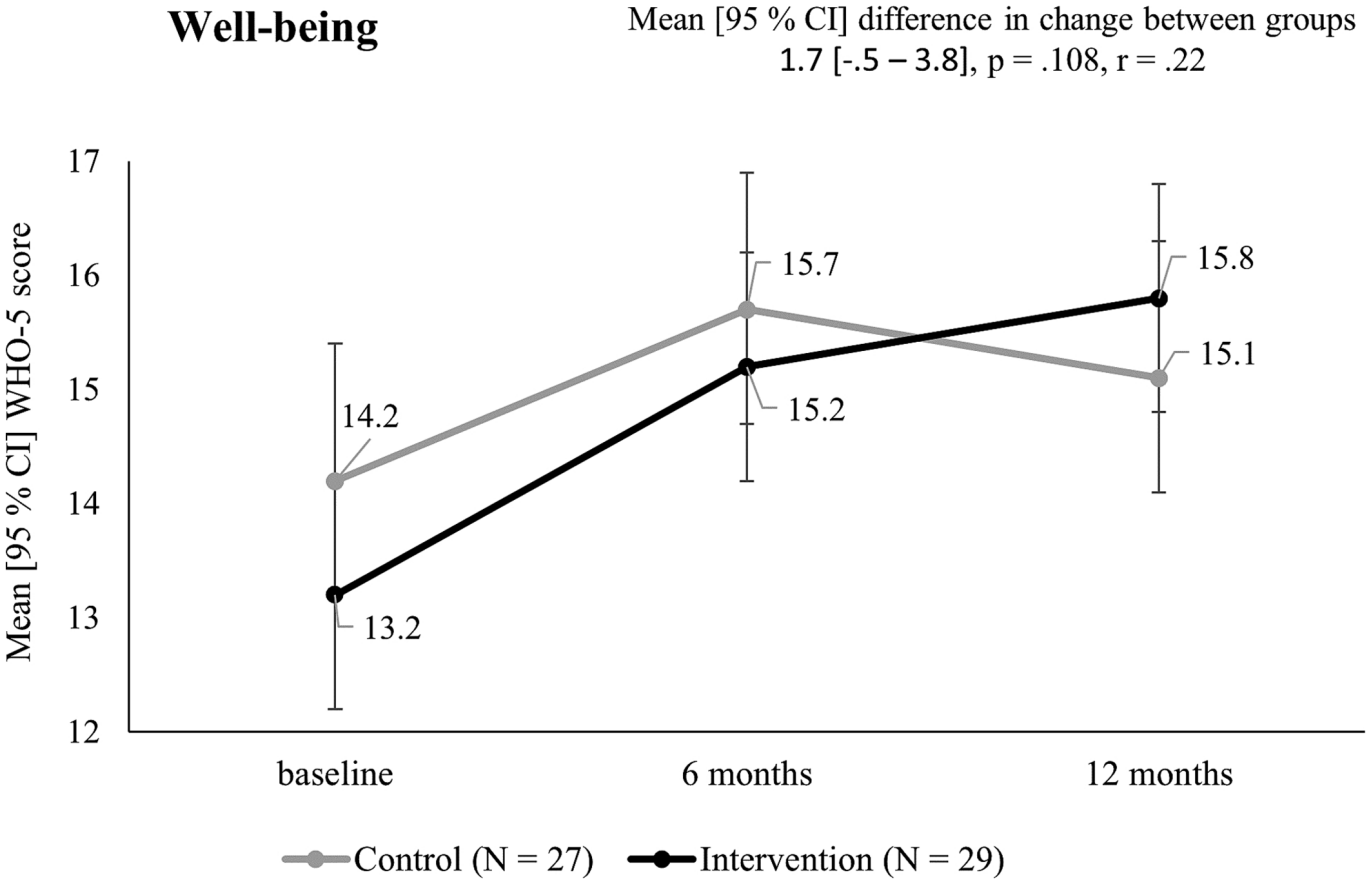
WHO-5 scores in the intervention and control group over the course of 12 months.

The difference in WHO-5 scores of IG [13.2 (5.1)] and CG [14.2 (3)] at baseline did not reach statistical significance (*p* = 0.36, *r* = 0.03). After 12 months, both groups improved. This resulted in a non-significant change of WHO-5 scores between groups of 1.7 [−0.5–3.8] (*p* = 0.108, *r* = 0.22). The change over time for both groups is displayed in Figure 5.

Mobility and strength values at t0, t1, and t2 as well as differences in change after 12 months (t0 – t2) between groups are presented in Table 1. Well-being values can be found in Table 2.

**Table 1:** Mobility and strength values over the course of 12 months in the intervention and control group.

	t0 (baseline)	t1 (after 6 months)	t2 (after 12 months)	Difference in change between groups after 12 months	*p*	*d*
FMS score^1^						
CG (*N* = 25)	10.7 (2.7)	10.2 (3.2)	10.6 (2.6)	6.3 [5.2–7.3]	< 0.001	3.3
IG (*N* = 28)	10.2 (2.6)	14.9 (2.5)	16.4 (2.4)			
TE (kg)						
CG (*N* = 25)	45.6 (17.4)	47.9 (17.8)	48.1 (18.9)	25.1 [18.9–31.2]	< 0.001	2.3
IG (*N* = 28)	52.5 (17.9)	73.3 (21.1)	80 (20)			
TF (kg)						
CG (*N* = 25)	35.1 (15.6)	34.5 (13.9)	33.1 (13.5)	19.6 [15.3–24]	< 0.001	2.5
IG (*N* = 28)	39 (15.6)	50.3 (17.5)	56.7 (19.2)			
TLFl (kg)						
CG (*N* = 25)	28.5 (12.6)	30.1 (11.8)	31.8 (13.3)	17.8 [13.7–21.9]	< 0.001	2.3
IG (*N* = 28)	32.8 (12.1)	48.5 (13.5)	54 (13.4)			
TLFr (kg)						
CG (*N* = 25)	29.5 (12.4)	30.4 (11.1)	33.3 (13.7)	16 [11.8–20.2]	< 0.001	2
IG (*N* = 28)	34.1 (12.6)	49.3 (13.3)	54 (13.8)			
UPush (kg)						
CG (*N* = 24)	60.9 (27.4)	61.6 (29)	63.1 (31.3)	26.9 [17.8–35]	< 0.001	1.7
IG (*N* = 28)	73.5 (34.1)	91.6 (41)	102.6 (43)			
UPull (kg)						
CG (*N* = 24)	52.6 (22.5)	53.8 (21.6)	53.8 (23.1)	13.2 [9.3–17.1]	< 0.001	1.9
IG (*N* = 28)	60 (24.7)	70.1 (25.6)	74.4 (26.3)			
HEl (kg)						
CG (*N* = 25)	38.2 (11)	43.3 (13.6)	44.4 (13.1)	16.9 [11.7–22.1]	< 0.001	1.8
IG (*N* = 28)	42.1 (14.4)	53.8 (16)	65.3 (15.7)			
HEr (kg)						
CG (*N* = 25)	43.7 (14.8)	41.1 (13.7)	41.3 (12)	17.2 [11.9–22.6]	< 0.001	1.8
IG (*N* = 28)	46.8 (14.5)	58.6 (16)	61.6 (16)			

Mobility (FMS score) and strength (kilograms) values at t0, t1, and t2 are expressed as mean (SD). Difference in change between groups is given as mean [95% CI]. Cohen’s d is given for effect size.

FMS, Functional Movement Screen; CG, control group; IG, intervention group; TE, trunk extension; TF, trunk flexion; TLFl, trunk lateral flexion left; TLFr, trunk lateral flexion right; UPush, upper body push; UPull, upper body pull; HEl, hip extension left; HEr, hip extension right.

^1^A score from 0 to 21 can be achieved.

**Table 2:** Well-being and back-issue values over the course of 12-months in the intervention (*N* = 29) and control group (*N* = 27).

	t0 (baseline)	t1 (after 6 months)	t2 (after 12 months)	Difference of change between groups after 12 months	*p*	*r*
WHO-5 score^2^						
CG	14.2 (3)	15.7 (4.4)	15.1 (4)	1.7 [−0.5–3.8]	0.108	0.22
IG	13.2 (5.1)	15.2 (4.5)	15.8 (4.8)			
Pain intensity^1^						
CG	3.8 (0.5)	3.4 (0.5)	3.3 (0.5)	−2.4 [−3.9 – -0.9]	0.003	0.4
IG	4.1 (0.5)	2 (0.5)	1.2 (0.4)			
Limitation^1^						
CG	3.3 (0.5)	2.9 (0.6)	1.9 (0.5)	−1.2 [−2.6–0.2]	0.13	0.2
IG	3.3 (0.5)	1.7 (0.4)	0.7 (0.3)			
Frequency^3^						
CG	2.9 (0.5)	2.7 (0.6)	2.7 (0.5)	−1.9 [−3.3 – -0.4]	0.009	0.35
IG	2.8 (0.5)	1.4 (0.4)	0.7 (0.3)			

Well-being (WHO-5 score) and back-issue values at t0, t1, and t2 are expressed as mean (SD). Difference in change between groups is presented as mean [95% CI]. Effect size *r* was calculated by dividing the z-value by the square root of the sample size N.

CG, control group; IG, intervention group.

^1^A score from 0 to 10 can be achieved.

^2^A score from 0 to 25 can be achieved.

^3^Frequency is given in days per week.

### 3.4 Exploratory endpoints

**Figure 6:**
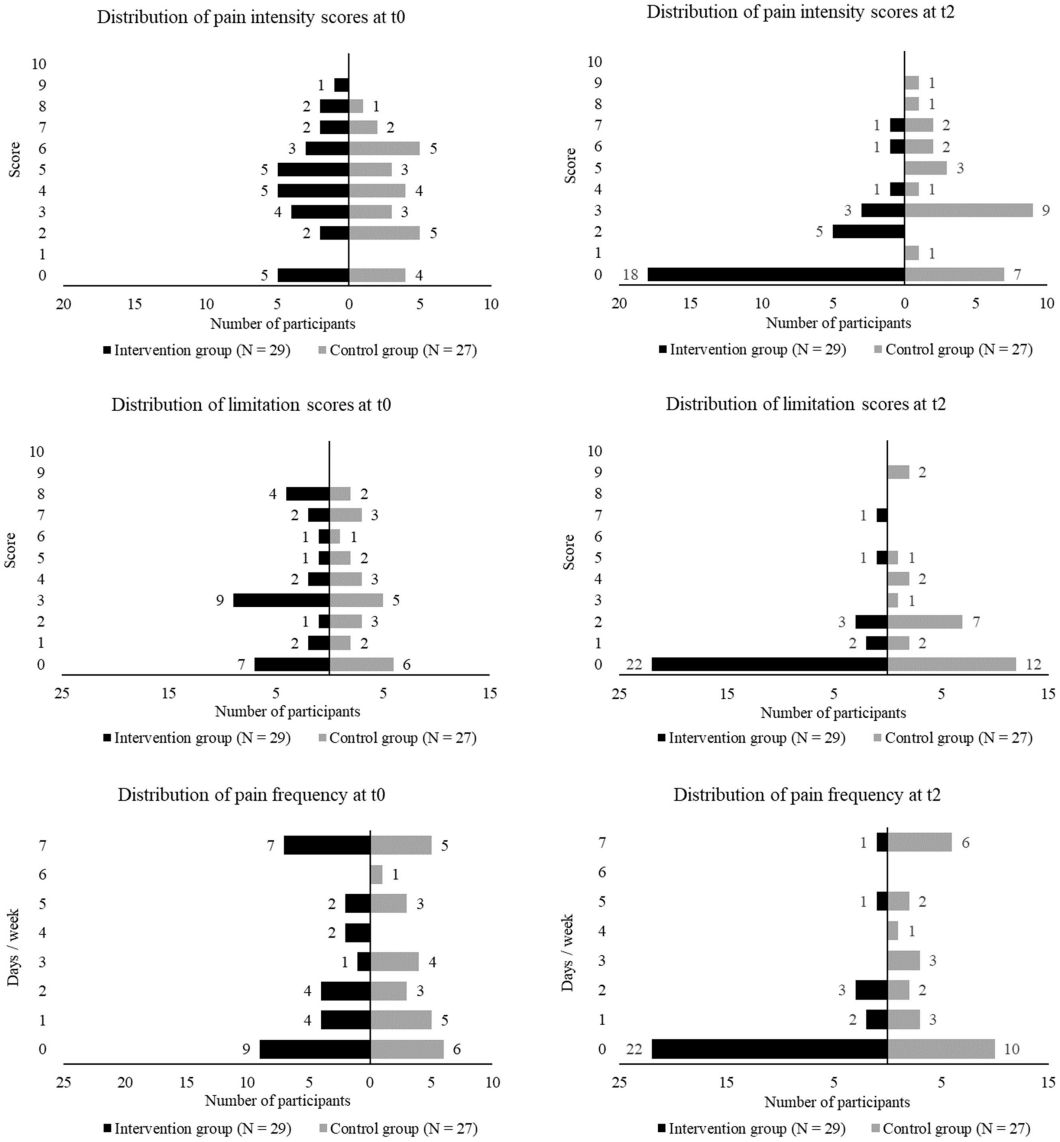
Distribution of back-issues (pain intensity, perceived limitation, pain frequency) at baseline (t0) and after 12 months (t2) in the intervention and control group.

IG and CG did not significantly differ in back-issue related pain intensity (*p* = 0.63, *r* = 0.06), perceived limitation (*p* = 0.97, *r* = 0.004), and pain frequency (*p* = 0.76, *r* = 0.04) at t0. After 12 months, the IG showed significantly greater reductions in pain intensity (−2.4 [−3.9 – -0.9], *p* = 0.003, *r* = 0.4) and pain frequency (−1.9 [−3.3 – -0.4], *p* = 0.009, r = 0.35) than the CG, but not in perceived limitation (−1.2 [−2.6–0.2], *p* = 0.13, *r* = 0.2) (Table 2). The proportion of pain-free participants reached 62.1% in the IG and 25.9% in the CG. A limitation score of 0 was reported by 75.9% of the IG and 44.4% of the CG. Pain frequency of 0 days / week was found in 75.9% of the IG and 37% of the CG. Distributions of back-issues at t0 and t2 are displayed in Figure 6.

## 4 Discussion

The results of the current study suggest that CrossFit® (*CF*) could be a successful workplace health intervention for physically inactive and sedentary employees. After 12 months, the intervention group (IG: N_t0_ = 55, N_t2_ = 29) showed large significant improvements in mobility (*p* < 0.001, *d* = 3.3) and maximum isometric strength (*p* < 0.001, minimum *d* = 1.7, maximum *d* = 2.5) compared to the control group (CG: N_t0_ = 34, N_t2_ = 28). Since a major challenge of workplace health promotion (WHP) is long-term participation, the success of *CF* was especially evident in terms of behavioral change and maintenance (33).

While previous research estimates 50% drop-out within the first 3–6 months of exercise programs we saw 47% in the IG and 18% in the CG after 12 months (34). Evaluation of dropout reasons underlines this aspect further. In the IG, only 9 of 15 participants that discontinued the program left for intrinsic reasons during the first 6 months (adherence 82%, non-adherence 15 percentage points). After another 6 months, 10 participants quit but only 1 of them for intrinsic reasons (adherence 74%, non-adherence 22 percentage points).

To identify internal and external factors that contributed to adherence, we analyzed participants’ capability, opportunity, motivation, and behavior based on the COM-B framework and the presence of behavior maintenance motives in *CF* training (13, 35).

As capability and opportunity are positively associated with physical activity behavior via the mediation effect of motivation, participation in WHP is less likely if these factors are lacking (36). At the UniBw M, physical and social opportunity were already advantageous prior the MedXFit-study. Employees had access to a comprehensive mix of aerobic-, strength-, as well as mobility-oriented courses and several training facilities on campus that were free of charge. The UniBw M supported physical activity by allowing employees to train 90 min per week during working hours and encouraged them to do so via e-mails, flyers, and information events. Adding *CF* to the portfolio changed neither physical nor social opportunity tremendously. Therefore, we assume that behavioral change and maintenance was mainly driven by increased capability.

Before the intervention, both groups showed low physical capability – based on mobility, strength, well-being, and back-issues. We assume that deficient physical capability negatively affected their confidence of being able to engage in physical activities. In contrast to other interventions on campus, we emphasized the suitability of *CF* training for individuals with poor fitness. Here we focused especially on the health-oriented approach as well as the high scalability of *CF* (14). Previous research suggests that simply knowing how to scale training in case of diminished physical capability (e.g., caused by injury, illness) minimizes barriers for participation in physical activities (37). As a result, we might have already positively affected participants before the intervention by giving them the feeling or belief of being capable (36).

Low physical capability was particularly evident in FMS scores of IG [10.2 (2.6)] and CG [10.7 (2.7)]. Such low mobility indicates that participants lacked the capability to perform fundamental movement patterns (e.g., squat, lunge, push-up) and were susceptible to injury, which is a major barrier to participation in physical activities (23, 24, 37). *CF* training consistently improved mobility in the IG compared to the CG. After 12 months, the mean FMS score of the IG [16.4 (2.4)] was higher than that of *CF* athletes with >60 months [15.2 (1.7)] and > 12 months [15.9 (2.4)] of *CF* experience (28, 29). A similar development was achieved by the IG for maximum isometric strength with improvements of 24–65% ((t2 – t0) / t0) within 12 months. Significant strength improvements from t1 to t2 in 6 out of 8 strength measures in the IG compared to the CG suggest that these effects occurred consistently and even in intermediates.

Improved capability was advantageous for motivation and behavior especially due to the performance-oriented training approach of *CF* – including scored workouts, competitions, execution of complex movements, tracking of training results, and striving for optimal fitness instead of just exercising. Capability (resp. fitness) is thus a measurable and perceivable prerequisite for participation as well as the main goal of *CF* (14). This implies close dependencies between capability, motivation, and behavior, from which the IG clearly benefited. At the beginning of the intervention, participants were directly confronted with their incapability during training. Even simple physical tasks such as standing up from the ground or deadlifting a medicine ball with good form were challenging for most participants. Throughout the training process, mobility and strength improvements gradually translated into healthier movement execution, greater training loads, better workout scores, and progression from simple to complex movements (e.g., from bodyweight squat to overhead squat to squat snatch). Achieving performance-related goals allowed participants to experience intrinsic motives such as enjoyment, challenge, and sense of competence that are typically found in sports and associated with behavioral maintenance (38, 39). Because *CF* is based on constant variation, participants could perceive their progress in a multitude of different exercises and workouts (14). Conversely, if participants stalled in one area (e.g., squat variation), the next training session already offered the chance to improve in another (e.g., handstand variation) making temporary stagnant performance less obvious and demotivating.

Regarding strength, largest improvements occurred in the trunk musculature which in turn could have benefited back-health (40–42). After 12 months, we observed a significant decrease in pain intensity (*p* = 0.003, *r* = 0.4) and frequency (*p* = 0.009, *r* = 0.35) in the IG compared to the CG. The percentage of pain-free participants in the IG increased from 17.2 to 62.1% (CG: 14.8% at baseline, 25.9% after 12 months). With injuries and illness being main barriers for physical activity participation, less back-issues could have supported behavior maintenance (37). Less pain an disability is also of interest in the context of WHP due to the high prevalence of sick leave caused by musculoskeletal disorders (15).

Well-being is another internal factor that independently increases the likelihood of long-term physical activity (43, 44). In this study, WHO-5 scores improved in both groups, leading to a non-significant change between groups (1.7 [−0.5–3.8], *p* = 0.108, *r* = 0.22). Interpretation of these results remains difficult as COVID-19 restrictions (e.g., remote work, decreased social interaction, access to recreational activities) might have interfered with positive effects of the additional physical activity (45).

**Figure 7:**
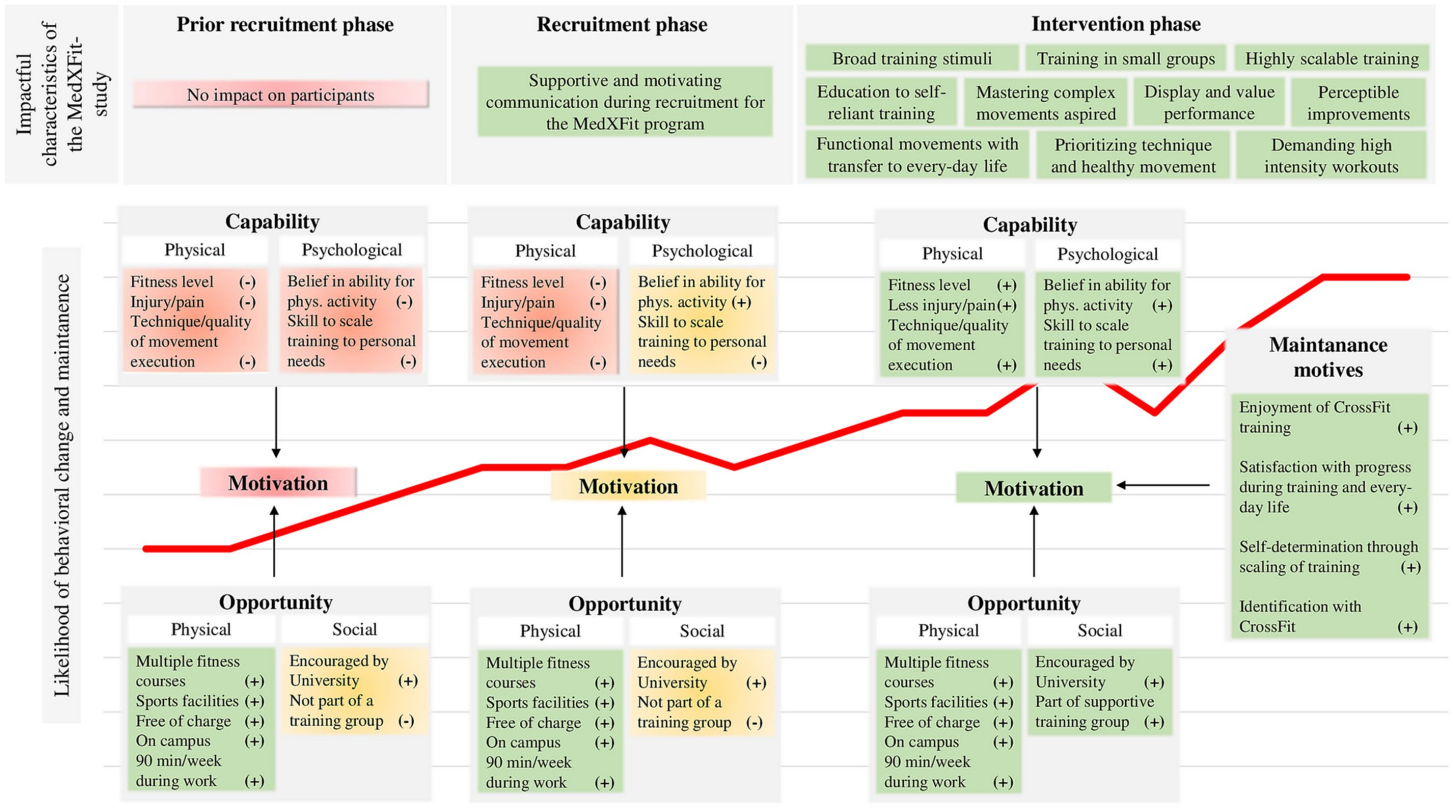
Likelihood of behavioral change and maintenance over the course of the MedXFit-study depending on capability, opportunity, motivation, and maintenance motives [cf. (13, 35)].

Nevertheless, behavioral change and maintenance did not rely purely on increased capability. Instead, group-based training also proved to be a key element for long-term motivation in multiple ways. In the MedXFit-study, young soldiers trained together with civilian employees, some of whom were about to retire. Such heterogenous groups made it imperative for participants to acquire the skill to modify training according to their fitness level. Knowing how to deal with deficient capability was found to be facilitator in the field of psychological capability (37). Additionally, self-regulating training by scaling volume, intensity, or movement selection offered a high degree of self-determination and therefore could have directly contributed to behavioral maintenance (35). In line with previous studies, we observed that social interaction among participants and coaches during training conveyed a great sense of affiliation (38). Over the course of the study, highly committed training partnerships and groups evolved which encouraged each other to show up for training. Moreover, participants assisted one another in training, cheered for their peers during workouts, bought *CF* training equipment and clothes, approached workouts more seriously, signed up for *CF* competitions, changed their diet, asked for additional skill training, and talked about *CF* related topics. In brief, they evolved from individuals doing *CF* to a community of CrossFitters that was motivated to keep training after studies end. This indicates the presence of several maintenance motives such as behavior enjoyment, satisfaction with behavioral outcomes, and congruence of the newly adopted behavior with their identity, beliefs, and values (35). Satisfaction with outcomes was further reflected in statements like: “I can easily carry my daughter now,” “I can walk without poles for the first time while hiking in the mountains over a gravel path,” or “I no longer have to ask my husband to carry the groceries from the car to our apartment but do it myself.” A schematic illustration of the likelihood for behavioral change and maintenance in dependence of capability, opportunity, motivation, and maintenance motives throughout the MedXFit-study is displayed in Figure 7.

As our analysis partly relies on observational data, findings regarding behavioral change and maintenance need to be verified by future research. Moreover, we would like to point out that the IG had higher strength values than the CG at baseline. Although these differences did not reach statistical significance, they should be considered when interpreting the results of the current study given the calculated effect sizes (minimum *d* = 0.21, maximum *d* = 0.41). Additionally, neither participants nor staff conducting the study was blinded and impact of COVID-19 on outcome variables remained unclear. Further lifestyle factors (overall physical activity, diet, alcohol consumption, sleep, and stress) should be documented in future research to better interpret outcomes. To quantify the potential of *CF* training in workplace settings, future studies should also measure sick days, productivity, and time expenditure for training related activities (e.g., transfer, training, hygiene) during working hours.

The WHP is challenged to identify interventions that are both effective in promoting health as well as motivating in the long-term. In this respect, the present follow-up study further extended the findings obtained after the first 6 months of the MedXFit-study published by Brandt et al. and thus contributed to the field of WHP (22). Previous research indicated that adaptations in the early phase of training programs are enhanced by motor learning and improved coordination but decline after the acquisition of basic motor skills (46). Since participants kept improving in health and fitness over the course of 12 months, we can now conclude that *CF* is also effective beyond the beginner level. To date, the current study is the only one concerning *CF* that allows to draw this conclusion for inactive, sedentary employees. Another important aspect we did not address before was behavioral change and maintenance, given the low participation and high dropout rates in WHP interventions (10–12). Therefore, unlike the evaluation of the first 6 months, the present study was supplemented by a comprehensive analysis of the participants’ behavior. In this regard, we observed that *CF* has excellent potential to induce behavioral change in previously inactive, sedentary employees. Additionally, we found important behavior maintenance motives such as behavior enjoyment, satisfaction with behavioral outcomes, self-determination, and congruence of the newly adopted behavior with one’s identity, beliefs, and values. Moreover, high scalability and versatile fitness adaptions allowed for training of heterogenous groups involving young soldiers as well as civilian employees shortly before retirement. Therefore, health professionals should be encouraged to consider *CF* for workplace health promotion.

## Data availability statement

The raw data supporting the conclusions of this article will be made available by the authors, without undue reservation.

## Ethics statement

The studies involving humans were approved by Institutional Ethics Committee of the University of the Bundeswehr Munich. The studies were conducted in accordance with the local legislation and institutional requirements. The participants provided their written informed consent to participate in this study.

## Author contributions

TB: Conceptualization, Data curation, Formal analysis, Investigation, Methodology, Visualization, Writing – original draft. EH: Investigation, Writing – review & editing. YK: Investigation, Writing – review & editing. SL: Investigation, Writing – review & editing. MM: Investigation, Writing – review & editing. TS: Investigation, Supervision, Writing – review & editing. AS: Conceptualization, Methodology, Supervision, Writing – review & editing.
