# Supplementary material for: The MedXFit-study – CrossFit as a workplace health intervention: a one-year, prospective, controlled, longitudinal, intervention study
Source: Front Public Health. 2024 Feb 21;12:1304721. doi: 10.3389/fpubh.2024.1304721 (PMC10915069; doi:10.3389/fpubh.2024.1304721)
Supplement: Supplementary file 1 [file Data_Sheet_1.PDF]

Table S1 Trial overview

Title	MedXFit-Study
Type	Prospective, controlled, intervention trial
Participants	N=89<br>• Self-selected groups<br>• Intervention group N=55<br>• Control group N=34<br>• Participants were not blinded
Study endpoints	Primary endpoints:<br>• Change in mobility (Functional Movement Screen score) from baseline to 12 months<br>• Change in mobility (Functional Movement Screen score) from 6 months to 12 months<br><br>Secondary endpoints:<br>• Change in maximum isometric strength from baseline to 12 months (including trunk extension, trunk flexion, trunk lateral flexion left, trunk lateral flexion right, upper body push, upper body pull, hip extension left, hip extension right)<br>• Change in maximum isometric strength from 6 months to 12 months (including trunk extension, trunk flexion, trunk lateral flexion left, trunk lateral flexion right, upper body push, upper body pull, hip extension left, hip extension right)<br>• Change in well-being (WHO-5 score) from baseline to 12 months<br>• Change in maximum isometric strength from 6 months to 12 months<br><br>Exploratory endpoints:<br>• Occurrence of back-issues (pain intensity, perceived limitation, pain frequency) in the neck, shoulder, upper back, and lower back at baseline, after 6 months, and after 12 months
Principal criteria	Inclusion criteria:<br>• 18 to 65 years of age (adults, older adults)<br>• Predominantly sedentary work<br>• Employed at the University of the Bundeswehr Munich (civilian and military)<br>• Physical inactive in term of less than 2 mobility and / or muscle enhancing training session per week<br>• Willingness to participate in CrossFit training<br><br>Exclusion criteria:<br>• Pregnancy<br>• Health issues that would disqualify for participation in regular exercise or the applied tests<br>○ severe injuries to the musculoskeletal system, osteoporosis, intervertebral disc damage, joint replacements, hypertension, fresh scars

Data collection	• Start of Study: October 2020<br>• End of Study: Dezember 2022<br>• Tests session were conducted at baseline (t0), after 6 months (t1), and after 12 months
Test session protocol	• All session followed the same protocol<br>1. Questionnaire (medical history / present health status, inclusion criteria, well-being, back-issues)<br>2. Body composition (weight, height)<br>3. Mobility assessment with Functional Movement Screen<br>4. Strength assessment with Dr. WOLFF BackCheck® 617<br>• All tests were conducted in sports wear without shoes<br>• Breathing mask (e.g., FFP-2) had to be worn during test sessions<br>• Participants avoided intensive physical training 24h prior test sessions
Body composition	• Height: SECA® 213<br>• Weight: TANITA® BC-545 scale
Mobility	Functional Movement Screen<br>• 7 fundamental movements (deep squat, hurdle step, inline-lunge, shoulder mobility, active straight leg raise, trunk stability push up and rotary stability quadruped)<br>• Shoulder mobility, trunk stability push up and rotary stability test contain a clearing test that identifies further pain<br>• Total score of 0 – 21 can be achieved<br>• Score per movement of 0 – 3 can be achieved<br>• 0 is given, when participants report pain during execution<br>• 1 – 3 is given dependent of movement execution quality<br>• For bilateral movements both sides were measured, the lower score was selected
Strength	Dr. WOLFF BackCheck® 617<br>• Maximum isometric strength in kg<br>• Conducted isometric tests: Trunk extension (TE), trunk flexion (TF), upper body push (UPush), upper body pull (UPull), trunk lateral flexion left (TLFl) and right (TLFr), and hip extension left (HEl) and right (HEr)<br>• 3 attempts per test, the best result was selected
Well-being	World Health Organization Well-Being Index (WHO-5)<br>• 5 items<br>• 5-point scale (1=worst, 5=best) for each item<br>• Total score ranges from 0 to 25
Back-issues	Questionnaire<br>• Participants self-reported pain intensity, perceived limitation, and pain frequency for the neck, shoulder, upper back, and lower back in the past 6 months<br>• Pain intensity and perceived limitation → 11-point scale (0=no pain/limitation, 10=highest imaginable pain/limitation)<br>• Pain frequency was reported in days per week
Type of intervention	Exercise → CrossFit training<br>• Twice a week for 60 minutes over the course of the study (12 months)<br>• Training conducted at the military affiliation CrossFit Kokoro<br>• No intervention for control group
