# Supplementary material for: The MedXFit-study – CrossFit as a workplace health intervention: a one-year, prospective, controlled, longitudinal, intervention study
Source: Front Public Health. 2024 Feb 21;12:1304721. doi: 10.3389/fpubh.2024.1304721 (PMC10915069; doi:10.3389/fpubh.2024.1304721)
Supplement: Supplementary file 2 [file Data_Sheet_2.PDF]

Table S2 Sample training sessions: Week 27 to 52

Sample training sessions for the training weeks 27 – 52 are provided in the table below.

The intervention group trained on 2 days per week. They self-selected the training day (Monday to Friday) but were encouraged to have at least 24 hours of rest between training sessions. Every training week was structured around a particular exercise - the main emphasis of the week. Nevertheless, training sessions differed daily. The training was done in groups (maximum 10 participants) under supervision of a certified CrossFit coach (CrossFit Level 1 and CrossFit Level 2). Although everyone followed the same program, the coach scaled volume, intensity, and movements according to the participants individual, physical condition.

Each training session lasted 60 minutes. The training session started with 5 - 10 minutes of introduction and warm up. Mobility was done afterwards for another 5 – 10 minutes, including the areas that are involved in the skill and strength part of the session. The skill and strength part lasted 5 – 30 minutes. After the skill and strength part, a high-intensity workout was performed for 5 – 30 minutes. Every session ended with 5 – 10 minutes of mobility and stretching.

Further explanations:

- E(x)MOM (y)= Every minute on the minute. Complete the prescribed repetitions per exercise in the given intervals and rest during the remaining time. When the given intervals differ from 1 minute, the x determines the length of intervals (e.g., E3MOM = every 3 minutes). When different movements are prescribed for the intervals, the sequence is written as 1) exercise 1, 2) exercise 2, etc. (y) determines the overall length.
- AMRAP = As many repetitions as possible are done in the given time span.
- For time = The prescribed workout is done as fast as possible.
- Tabata = 8 consecutive sets of 20sec work followed by 10sec rest. When different movements are prescribed for the intervals, the sequence is written as 1) exercise 1, 2) exercise 2, etc..
- (x)-(x)-(x)-(x) repetitions = Complete x repetitions of every exercise as fast as possible. 21 – 15 – 9 Pull ups, Thruster = Complete 21 pull ups, 21 thruster, 15 pull ups, 15 thruster, 9 pull ups and 9 thruster as fast as possible.
- Technique = Technique training of complex movements. Intensity was kept low.

	Main emphasis	Warm-up	Mobility	Skill and strength	"Workout of the day"	Cool-down
Week 27	Dumbbell clean and press	3 Rounds<br>10 Good morning<br>10 Squat<br>10 Slow mountain climber<br>10 Dumbbell row	Ankle, hip, wrist	E3MOM 21<br>3-5 Rounds dumbbell complex<br>1 Dumbbell power clean + 2 Dumbbell front rack squat + 3 Dumbbell push press	10min AMRAP<br>2 Slamball thruster<br>2 Sit up<br>4 Slamball thruster<br>4 Sit up<br>*add 2 repetitions each round*	Mobility / stretching

Week 28	Rowing ergometer	20m Walking lunge<br>15 Sit up<br>20m Jog<br>15 Squat<br>20m Walking lunge<br>15 Push up<br>20m Jog<br>15 Good morning	Ankle, shoulder, thoracic spine	(1) E4MOM 20<br>20cal Row + 20 Swimmer + 20 Sit up<br><br>(2) Tabata<br>Wall angel<br>Hollow hold	9min AMRAP<br>20 High knee<br>50m Run<br>10 Burpee<br>50m Run<br>15 Jumping jack	Mobility / stretching
Week 29	Pull up	5 rounds Slamball complex<br>5 Deadlift<br>5 SlamBall clean<br>5 Front squat<br>5 Press<br>5 Slamball	Shoulder, thoracic spine	(1) EMOM 21<br>1) 5-10 Pull Up / jumping pull up / Australian pull up<br>2) 5-10 Push up<br>3) 5-10 Split squat<br><br>(2) Tabata<br>1) Side plank pulses right<br>2) Side plank pulses left	For time<br>10 Slamball thruster<br>15 Burpee<br>20 Slamball thruster<br>15 Burpee<br>30 Slamball thruster<br>15 Burpee<br>40 Slamball thruster<br>15 Burpee<br>50 Slamball thruster<br>15 Burpee<br>60 Slamball thruster<br>15 Burpee<br><br>Time cap: 18 minutes	Mobility / stretching
Week 30	Dumbbell snatch	5min Loaded carries with dumbbells (farmers, waiters, suitcase, etc.)	Hip, shoulder	(1) Dumbbell snatch technique<br>Dumbbell deadlift<br>Dumbbell pull<br>Dumbbell high pull<br>Dumbbell muscle snatch<br>Dumbbell snatch<br><br>(2) Tabata<br>1) Seated leg lift<br>2) Swimmer	EMOM 10<br>5 Burpee + 20 Single under<br>*then*<br>50 goblet Squats	Mobility / stretching

Week 31	Shoulder press	2 rounds<br>10 Push up<br>10 Bird dog<br>10 Push up<br>10 Inchworm	Hip, shoulder, thoracic spine	(1) Barbell shoulder press technique<br><br>(2) 5 supersets<br>8 Barbell press<br>8 Scapular pull up / band pull apart<br><br>(3) Tabata<br>1) Side plank pulse right<br>2) Side plank pulse left	7min AMRAP<br>Burpee	Mobility / stretching
Week 32	Squat	10 World's greatest stretch<br>10 Air squat<br>10 Push up<br>10 Knee hug	Hip, ankle	(1) DB Goblet squat technique<br><br>(2) EMOM 18<br>1) 10 DB Front hold squat<br>2) 10 Australian pull up<br>3) 30sek Plank	For time<br>10 Burpee<br>50m Run<br>20 Sit up<br>50m Run<br>30 Lunge<br>50m Run<br>40 Air squat<br>50m Run	Mobility / stretching
Week 33	Snatch	3-5 rounds Slamball complex<br>5 Deadlift<br>5 Slamball clean<br>5 Front squat<br>5 Slamball<br>5 Push up	Ankle, hip, shoulder	(1) Snatch technique<br>Snatch grip deadlift<br>Snatch grip high pull<br>Snatch grip press behind the neck<br>Overhead squat<br>Drop snatch<br>Snatch from hip height<br><br>(2) Tabata<br>1) Side plank pulse right<br>2) Side plank pulse left	3 Rounds for max repetitions<br>1 Min Wall ball shot<br>1 Min Slamball<br>1 Min Box jump<br>1 Min Push up<br>1 Min Max cal row<br>1Min Rest	Mobility / stretching
Week 34	Pull ups	3 Round<br>5 Scapular Pull up<br>5 Scapular Push up<br>10 Overhead squat	Shoulder	(1) EMOM20 (30sec work/30sec rest)<br>1) Jumping pull up<br>2) Slamball thruster<br>3) TRX row<br>4) Barbell thruster<br><br>(2) Tabata<br>1) Hollow hold<br>2) Superman	12min AMRAP<br>2 Lunge<br>2 Russian twist (2-count)<br>4 Lunge<br>4 Russian twist (2-count)<br>*add 2 repetitions each round	Mobility / stretching

Week 35	Snatch	2 rounds<br>10 Push Ups<br>10 Kettlebell Deadlift<br>15m Suitcase Carry<br>10 Kettlebell Front hold lunge<br>15m Front hold Carry<br>10 Kettlebell Press<br>15m Overhead carry	Hip, shoulder	(1) Snatch technique<br>Muscle Snatch<br>Overhead squat<br>Behind neck snatch grip press<br>Behind neck snatch grip sots press<br>Drop snatch<br><br>(2) Tabata<br>1) Seated leg lift<br>2) Tuck hold	For time<br>21-15-9<br>One arm dumbbell overhead squat<br>Burpee box jump over	Mobility / stretching
Week 36	Deadlift	200m run<br>8 Push up<br>16 Squat<br>22 Dead bug<br>16 Squat<br>8 Push up<br>200m run	Ankle, hip, shoulder	(1) Deadlift 5 RM<br><br>(2) Sumo deadlift high pull 5 RM	For time<br>3-9-15-21-15-9-3<br>Deadlift (Rx 93/ 66 kg)<br>Box jump (24/20 inch)	Mobility / stretching
Week 37	Russian Kettlebell Swing	3 Rounds<br>10 Deadlift<br>10 Row<br>10 Lunge<br>10 Goblet squat<br>10 Push up	Hip	(1) Kettlebell swing technique<br><br>(2) Tabata<br>1) Side plank left<br>2) Side plank right	For time<br>10-9-8-7-6-5-4-3-2-1<br>Burpee box jump<br>Kettlebell swing	Mobility / stretching
Week 38	Overhead stability	2 Rounds (each side)<br>1 Turkish get up<br>5 Windmill<br>5 Kneeling one-arm press<br>10 Row<br>10 Overhead lunge	Hip, shoulder, thoracic spine	E3MOM27<br>5-10 Scapula pull up + 5 Overhead squat<br>2sec:2sec:2sec:2sec (down: hold: up: hold)	10min AMRAP<br>10 Kettlebell high pull<br>5 Burpee	Mobility / stretching
Week 39	Rowing erg	2 Rounds<br>5 Swimmer<br>10 Wall angel<br>15 Sit up<br>20 Air squat	Hip, shoulder, thoracic spine	(1) EMOM 24<br>1)7cal Row<br>2) 15 Sit up<br>3) 10 Thruster<br>4) 10 Ring row<br><br>(2) Tabata<br>Hollow hold	For time<br>20cal Row<br>*then*<br>3 Rounds<br>20 Deadlift<br>30 Box jump	Mobility / stretching

Week 40	Dumbbell Snatch	3 Rounds (with kettlebell)<br>10 Deadlift<br>10 Goblet squat<br>10 Swing<br>1 Turkish get up each side	Hip, shoulder, thoracic spine	(1) Dumbbell snatch technique<br><br>(2) Hanging knee raises technique	20min AMRAP<br>8 Hanging knee raise<br>10 Dumbbell snatch<br>14ca. Row	Mobility / stretching
Week 41	Pull up, Thruster	2 Rounds<br>10 Snatch grip deadlift<br>10 Snatch grip high pull<br>10 Snatch grip press<br>10 Overhead lunge<br>10 Overhead squats	Ankle, hip, shoulder, wrist	(1) Muscle clean + push press technique<br><br>(2) E2MOM 16<br>Complex 1 Muscle clean, 2 front squat, 3 push press<br><br>(3) Tabata<br>Sit up	"FRAN"<br>21-15-9<br>PullUps<br>Thruster	Mobility / stretching
Week 42	Deadlift	5 Rounds<br>10 Wall Facing quat<br>10 Wallball shot	Hip, upper back	(1) E3MOM 24<br>5 Deadlift (increase weight each round)<br>10 Ring row<br><br>(2) Tabata<br>Hollow hold	For time<br>21-15-9<br>Deadlift 70% of 5 RM<br>Dumbbell thruster	Mobility / stretching
Week 43	Back Squat	5min Loaded carries with dumbbells (farmers, waiters, suitcase, etc.)<br><br>3 Rounds<br>1 Turkish get up (each side; 10sec up, 10sec down)<br>10 Slow wall facing squat	Ankle, hip	(1) Back squat technique<br><br>(2) E3MOM 21<br>5 Back squat + 10 dumbbell row	For time<br>5 Rounds<br>10 Deadlift<br>10 Burpee	Mobility / stretching
Week 44	Overhead press	3 Rounds<br>200m Run<br>10 Burpee<br>15m Inch worm	Shoulder	Overhead press<br>Build up to 5 RM	For time<br>5 Rounds<br>10 Shoulder to overhead (100% of strict press 5RM)<br>10 Toes to bar / knees to elbow	Mobility / stretching
Week 45	Pull up	3 Rounds<br>10 Walk out<br>10 Sit up<br>10 Burpee	Shoulder	(1) EMOM 10<br>3 Pull up<br><br>(1) Tabata<br>1) Hang from bar in hollow position<br>2) Hang from bar in arched position	AMRAP 20<br>20cal bike<br>15 Push up<br>10 Dumbbell snatch<br>5 Sit up	Mobility / stretching

Week 46	Front squat	3 Rounds Kettlebell complex<br>10 Deadlift<br>10 Goblet squat<br>10 Swing	Ankle, hip, shoulder, wirst	E2MOM 18<br>5 Paused front squat (3sec) + 10 ring row	AMRAP 20<br>15 Box jump<br>12 Shoulder to overhead<br>9 Toes to bar	Mobility / stretching
Week 47	Handstand	3 Rounds<br>10 Walkout<br>8 Sit up<br>6 Lunge<br>4 Hollow rock<br>2 burpee	Shoulder, thoracic spine	Handstand technique	For time<br>100 Squat<br>80 Lunge<br>60 Jumping squat<br>40 Jumping lunge<br>20 Pistol squat	Mobility / stretching
Week 48	Rowing ergometer	3 Rounds barbelll complex<br>15 Deadlift<br>15 Row<br>10 Press<br>15 Squat	Shoulder, hip	EMOM 20<br>1) 10 Shoulder press<br>2) 15 Kettlebell swing<br>3) 15 Sit up<br>4) 15 Barbell squat<br>5) 30sec Single under	For Time<br>500m Row<br>*Rest 1min*<br>500m Row<br>*Rest 1min*<br>500m Row	Mobility / stretching
Week 49	Back squat	3 Rounds<br>10 Air squat<br>10 Inch worm<br>10 Russian twist	Ankle, hip	E3MOM 15<br>10 Back squat	For Time<br>60cal Row<br>40 Wallball shot<br>100 Single under	Mobility / stretching
Week 50	Push press, push jerk	3 Rounds<br>10 Dumbbell deadlift<br>10 Dumbbell row<br>10 Dumbbell press<br>20m Dumbbell overhead carry	Shoulder, thoracic spine, wirst	(1) Push press, push jerk technique<br><br>(2) E2MOM 14<br>3 Push Press (hold overhead position for 5sec)	For time<br>1000m Row<br>50 Thruster<br>50 Kettlebell Swing	Mobility / stretching
Week 51	Thruster	2 Rounds<br>10 Clean grip deadlift<br>10 Clean grip highpull<br>10 Clean grip press<br>10 Front rack lunge<br>10 Front squat	Shoulder, hip	E2MOM 20<br>5 Thruster (hold bottom position 3sec)	AMRAP 20<br>250m Row<br>10 Thruster<br>10 Sit up<br>10 KB Swing	Mobility / stretching
Week 52	Double under	2 Rounds<br>15cal Row<br>15 Jumping jack<br>5 Inch worms	Ankle, wrist	Double under technique	For Time<br>100-80-60-40-20<br>Air squat<br>Mountain climber<br>Double under<br>*every 2min 5 burpee*	Mobility / stretching
